# Elimination of artifacts caused by residual radiopharmaceutical activity in injection site in myocardial perfusion imaging on Discovery NM 530c semiconductor gamma camera

**DOI:** 10.1186/s13550-025-01222-w

**Published:** 2025-03-28

**Authors:** Aleksandra Owczarek, Zbigniew Adamczewski, Anna Plachcinska, Pawel Cichocki

**Affiliations:** Department of Nuclear Medicine, Medical University, Lodz, Poland

**Keywords:** Artifacts, CZT SPECT, Myocardial perfusion imaging

## Abstract

**Background:**

Cardiac gamma cameras dedicated for myocardial perfusion imaging (MPI) perform studies faster and acquire higher quality images than traditional cameras. However, they are susceptible to some artifacts. We observed a previously unreported artifact, caused by residual radiopharmaceutical activity in injection site in cubital fossa caught in camera field of view. This study aims to assess the impact of these artifacts on image quality and the possibility of their elimination. Study included 50 male patients referred for MPI using Discovery NM 530c gamma camera, in whom radiopharmaceutical activity in injection site was observed in stress or rest study. In such cases, image acquisition was immediately repeated, with the patient and the camera kept in the same position, after covering the injection site with a special lead shield. Obtained images were assessed by two experienced nuclear medicine physicians using a 0–4 point scale in each segment (where 0—normal perfusion, and 4—complete lack of perfusion). Summed stress, rest and difference scores (SSS, SRS and SDS, respectively) were calculated for the entire myocardium and 3 main vascular territories.

**Results:**

SSS, SRS and SDS were most frequently assessed as abnormal in RCA territory. Radiopharmaceutical activity in injection site was observed more frequently in stress studies (84% of cases). Covering injection site with a shield changed the assessment of SSS, SRS or SDS from normal to abnormal and vice versa in almost 20% of studies. The most frequently affected vascular territories were LAD and RCA. Elimination of the artifact changed final diagnosis in almost 1/5 of patients, most often by eliminating previously visible significant stress-induced perfusion defects (patients in whom such change occurred did not report any cardiovascular events in one-year follow-up).

**Conclusions:**

Artifacts caused by radiopharmaceutical activity in injection site reduce image quality and can potentially generate or hide perfusion defects. They can be observed mainly in patients examined in prone position, after radiopharmaceutical injection in cubital fossa. These artifacts can be eliminated by a lead shield, which can change the final assessment of MPI study in 20% of the patients.

**Supplementary Information:**

The online version contains supplementary material available at 10.1186/s13550-025-01222-w.

## Background

Coronary artery disease (CAD) is characterized by insufficient blood supply to the heart muscle due to partial stenosis or complete blockage of the coronary arteries supplying blood to the myocardium. Impaired blood flow through a coronary artery may be caused by cholesterol deposits (due to atherosclerosis), clot (due to existing thrombosis, trauma, or other heart diseases), compression by a muscle bridge, or vessel spasm (Prinzmetal’s angina). CAD is one of the most common causes of death in developed countries [1]. It is estimated that in most European countries median prevalence of CAD in 2019 was 2895 people per 100 000 inhabitants [2]. In order to avoid serious complications of CAD, such as myocardial infarction, a patient with suspected CAD should be referred for appropriate studies. Nowadays, diagnostic imaging of CAD includes such techniques as ultrasound echocardiography, computed tomography and magnetic resonance imaging, as well as single photon emission computed tomography (SPECT) and positron emission tomography (PET) [3]. One of the frequently chosen modalities in diagnosis of CAD is myocardial perfusion imaging (MPI) employing the SPECT technique [3–5]. Currently, gamma cameras equipped with one of two types of detectors—scintillation crystals and semiconductors—are used to perform this study, with the latter characterized by better accuracy and sensitivity in detecting CAD [6]. Despite the introduction of advanced semiconductor technology [7, 8], even newest models of gamma cameras can still generate artifacts that negatively affect the assessment of images [9–12].

One kind of such artifacts, that has not been reported yet, could be observed in MPI studies performed using the dedicated cardiac semiconductor gamma camera GE Discovery NM 530c (GE Healthcare, Chicago, IL, USA), as a result of small residual activity of the radiopharmaceutical remaining after intravenous administration in the area of the patient’s elbow bend and located at the edge of the camera field of view. This study aims to investigate the impact of these artifacts on the quality of the reconstructed images and to assess the possibility of their elimination with a lead shield.

## Methods

The study consisted of patients of the Department of Nuclear Medicine at the Central Teaching Hospital of the Medical University of Lodz referred for a routine [^99m^Tc]Tc-MIBI MPI performed using cardiac gamma camera Discovery NM 530c (GE Healthcare, Chicago, IL, USA), in total approximately 600 patients examined from July 2022 to June 2023. MPI was performed in standard, two-day, stress-rest protocol used in our Department, based on European Nuclear Medicine Association guidelines [13]. All studies were performed using [^99m^Tc]Tc-MIBI injected intravenously via a 0.9 × 25 mm catheter. Patients referred for static MPI received 3 MBq/kg b.m. administered in a seated position, while patients referred for MPI with additional dynamic coronary flow reserve assessment received constant activity of 587 MBq administered in supine position on the gamma camera bed. Static studies in both cases were carried out approximately one hour after radiopharmaceutical injection. Male patients were being examined in prone position, while female patients—in supine position. During static image acquisition in some patients, spots of activity, located in the area of the right forearm or elbow, were observed in the gamma camera field of view. Their position corresponded to the radiopharmaceutical injection site. Presence of such spots of activity was the criterion for including the patient in the study. Patients in whom other types of artifacts (caused by patient motion or significant extracardiac activity of the radiopharmaceutical) were also observed were excluded from the study.

In total, the study included 50 male patients aged 40 to 83. Full demographic and clinical data of the patients is included in supplementary materials. All static MPI studies of these patients were performed in prone position, with the patient’s arms and head placed on a dedicated support provided by camera manufacturer.

**Fig. 1:**
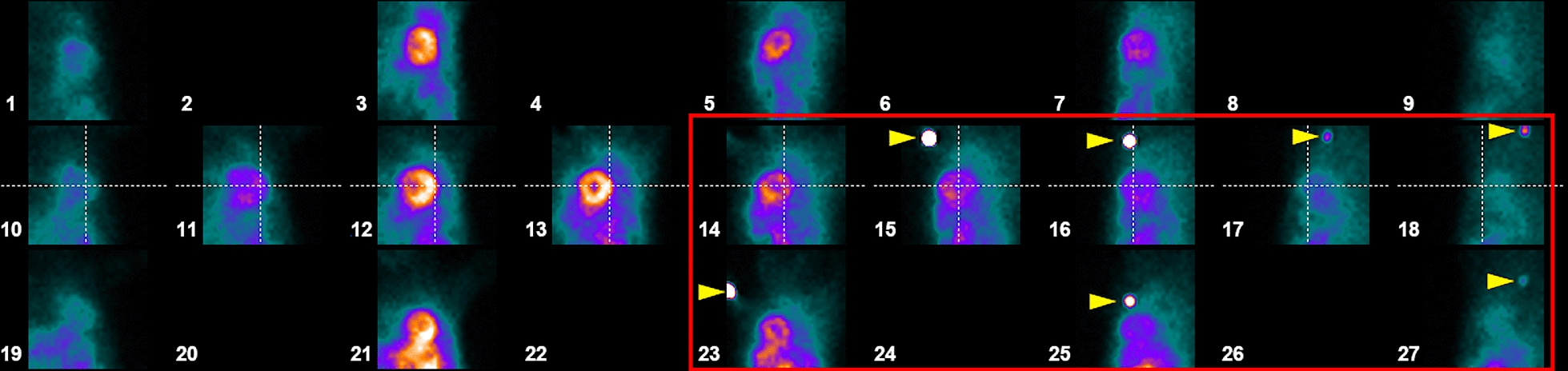
Location of the artifact in images displayed in Xeleris workstation, „Scan QC” tab. In examined material, spots of activity remaining at the site of radiopharmaceutical administration were typically visible in one or several projections from the ones marked with the red frame (spots visible in this patient are marked with yellow arrows)

After acquiring images, but before moving the camera bed and releasing the patient, the technician checked the images using the Xeleris workstation for the presence of activity at the site of radiopharmaceutical administration. The artifact appeared as a spot of activity located in the upper parts of planar images obtained from one or several detectors acquiring images of the heart in the posterior and lateral projections, marked with numbers 14–18, and 23–27 (Fig. 1). The artifact was most often observed in projection no. 15. Throughout the evaluation of the images, the patient constantly remained on the gamma camera bed in the same position and with the same detector positioning as during the acquisition of the images.

**Fig. 2:**
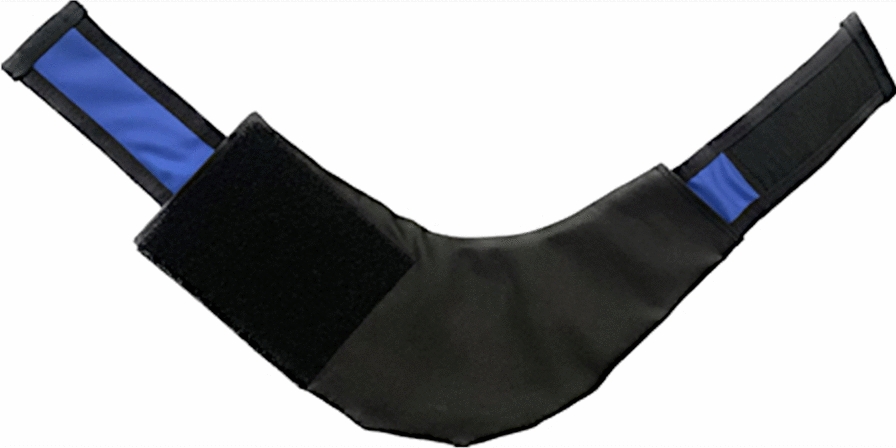
Reinforced lead thyroid shield used in the study to cover the injection site

**Fig. 3:**
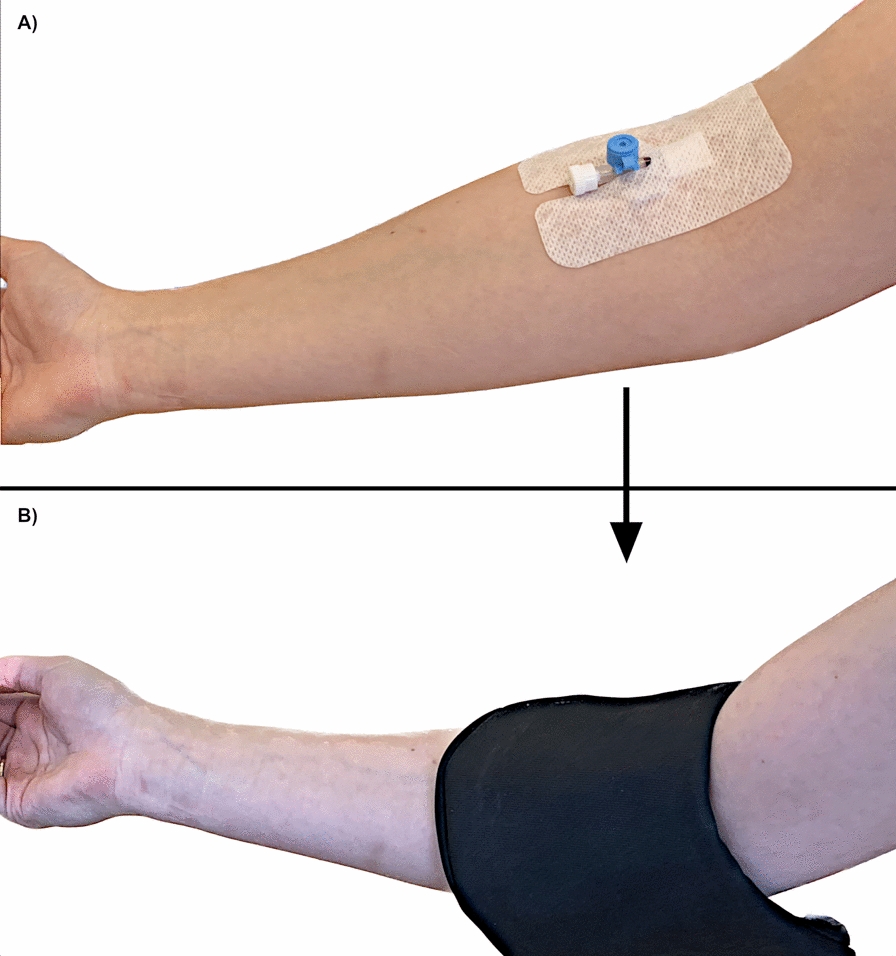
Example of shield positioning (**B**), covering the site of radiopharmaceutical injection (**A**)

When radiopharmaceutical activity was observed at the injection site, the technician performing the study was immediately repeating image acquisition, after covering the injection site with a specially prepared lead shield, based on a medical shield designed to protect the thyroid gland in classic radiography. The shield was additionally reinforced with a layer of lead sheet, to a total lead equivalent thickness of approx. 2.5 mm (Fig. 2). This thickness of the material was tested earlier and it was considered sufficient to absorb the radiation emitted by the technetium activity that typically remained in the injection site. The additional lead sheet was covered with protective material, separating it from the skin of a patient, and a fastener has been added to the distal parts of the cover to make it easier to fix it in the right place, consistently covering the injection site during image acquisition. Example of the shield positioning is presented on Fig. 3.

MPI with the shield was performed using the same image acquisition protocol as before. Patient and detector positioning also remained the same.

Acquired images were segregated into two sets:

• set I—base stress/rest images, with the artifact (without shield).

• set II—stress/rest images with repeated study (after applying the shield).

**Fig. 4:**
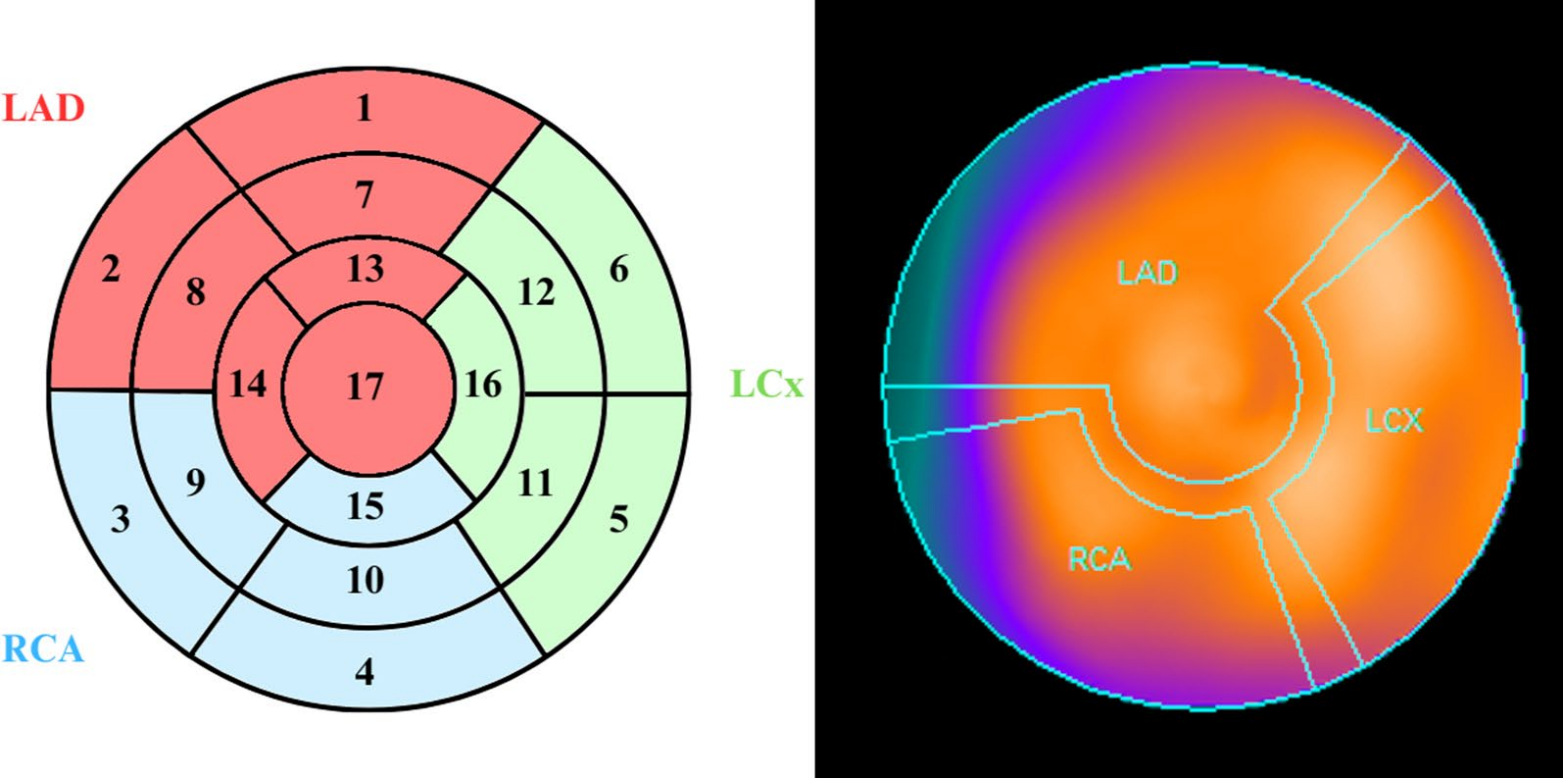
On the left side—a simplified diagram of the 17-segment left ventricle model, with areas typically supplied by the three main coronary vessels marked in different colors. On the right side—a polar map of the heart with analogous vascular territories

Both sets were visually assessed by two nuclear medicine specialists to check the clinical significance of observed artifacts, without relying on any automated software-based calculations [14]. Each study was assessed according to the 17-segment model of the left ventricle (Fig. 4), using a standard 5-grade scale (where 0—normal perfusion, and 4—complete lack of perfusion) by consensus. Summed rest, stress and difference scores (SRS, SSS and SDS, respectively) were calculated for the whole myocardium, as well as for individual vascular territories (LAD, LCx and RCA), based on the grades applied to each segment in rest and stress studies. First, all studies from set I were assessed, then, after approximately 2 weeks, studies from set II were assessed. Then, differences in scores between both sets of each patient were calculated.

However, it has to be noted that discrepancies between the point values in studies with and without the shield are not always significant from a clinical point of view. In practice, only those differences that change the image classification in terms of presence or absence of significant stress-induced ischemia are important, which may influence the final diagnosis of CAD and further treatment of the patient. Therefore, differences in the evaluation of the studies were also analyzed in this respect, with SDS ≥ 3 for the entire myocardium and SDS ≥ 2 for individual vascular territories used as cutoff values for significant stress-induced ischemia.

## Results

Activity in injection site was observed in both rest and stress studies, however it was much more common in stress studies—84% of patients. In rest studies, this activity was observed in only 16% of patients. In the examined material, the artifact was observed only in one of the two studies in each patient (either in rest or stress study, not in both). In rest studies, the site of injection was located in the right cubital fossa in 100% of cases. In stress studies, injection site was also most commonly located in the right cubital fossa—96% of cases, while in 2 patients (4%) it was in the forearm. In studies in which the catheter was in locations other than the right forearm or cubital fossa, the artifact was not observed.

For the whole myocardium, comparing studies with and without the shield, SRS or SSS values changed in as many as 70% of cases, and SDS values in 60%, although these differences may be partially attributed to operator-related variability in independent assessments of each set of studies. For individual vascular territories, such change occurred in LAD in 38% (SDS—32%), in LCx in 20% (SDS—18%), and in RCA in 46% (SDS—32%) of cases. All values are summarized in the Table 1. Values for each patient are included in supplementary materials.

**Table 1:** Differences in SSS, SRS and SDS values after application of the shield (compared to studies without the shield) for the whole myocardium (TOT) and individual vascular territories

	TOT			LAD			LCx			RCA		
	SSS	SRS	SDS	SSS	SRS	SDS	SSS	SRS	SDS	SSS	SRS	SDS
No change	12	3	20	24	7	34	34	6	41	22	5	34
Lower score	17	4	16	8	1	8	5	2	6	15	2	11
Higher score	13	1	14	10	0	8	3	0	3	5	1	5

**Fig. 5:**
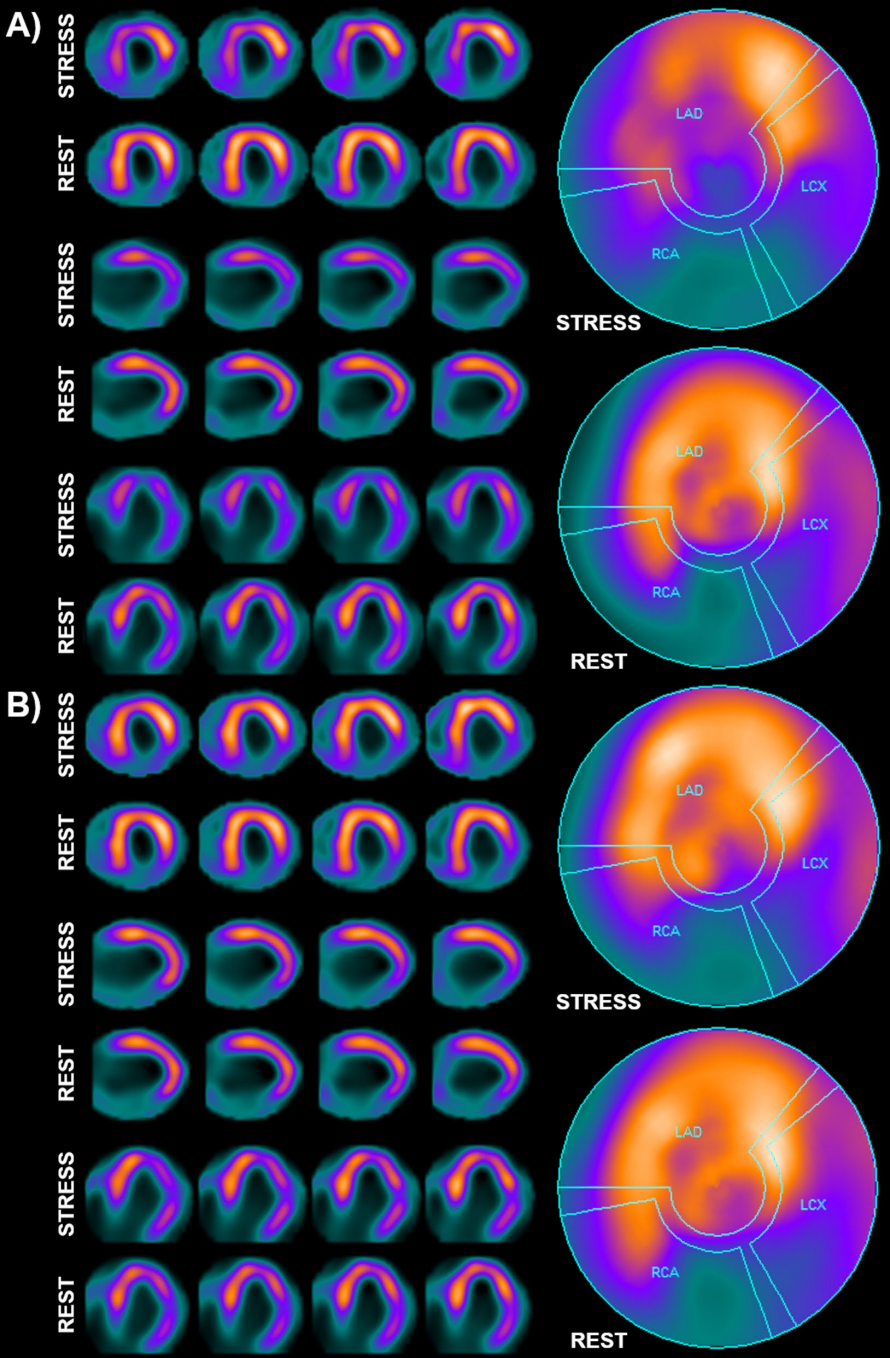
**A**—base set of images with an artifact present in the stress study. Clinical evaluation revealed features of significant stress-induced ischemia in the apex and interventricular septum (SDS = 10, qualifying the patient for coronary angiography) and permanent perfusion defect in the inferior wall (post-infarction scar). **B**—second set of images, with stress study repeated with the shield. Clinical evaluation revealed only features of post-infarction scar in the inferior wall, with no features of stress-induced ischemia (SDS = 0, no indications for invasive diagnosis)

Despite such frequent discrepancies between SRS and SSS values in studies with and without the shield, not all differences were significant from a clinical point of view. For the whole myocardium, changes in the final assessment of the image as normal/abnormal occurred in almost 20% of cases. For individual vascular territories, the elimination of the artifact changed the diagnosis in over 10% of the examined vessels. It should be noted that the occurrence of the artifact only in the rest study did not influence the final diagnosis regarding the presence or absence of stress-induced ischemia in any of the assessed patients. The results are summarized in the Table 2, and an example of a clinically significant difference between study sets with and without the shield is shown on Fig. 5.

**Table 2:** Differences after shield placement in the presence or absence of significant stress-induced ischemia in the whole myocardium (TOT) and individual vascular territories

	TOT	LAD	LCx	RCA
Without changes	41	44	46	44
Induced ischemia → no ischemia	7	4	3	5
No ischemia → induced ischemia	2	2	1	1

Among 8 cases where the artifact was observed in rest studies, in 3 patients who had no history of infarction and normal contractility in echo the initial SRS of 2–5 was reduced to 0 after applying the shield, which better aligns with clinical data.

Follow-up clinical information of cardiovascular events was obtained from 45/50 patients approximately 1 year after MPI study (5 patients did not respond to calls). 7/45 patients were hospitalized due to cardiovascular events (3 underwent coronary angioplasty, 1—coronary artery bypass graft surgery, in 2 coronary angiography revealed no significant stenosis, and 1 underwent only conservative treatment). Additionally, 4 other patients reported incidents of arrythmias and atrial fibrillation (1 treated with ablation). Remaining 34 patients reported no cardiovascular events.

Among patients in whom applying the shield changed final MPI report in terms of presence or absence of significant stress-induced ischemia, none was hospitalized due to cardiovascular events (this includes 5 patients in whom applying the shield changed the study result from abnormal to normal and 2 in whom it revealed previously undetected ischemia).

## Discussion

Due to great importance of MPI in the diagnosis of CAD, it is vital to ensure the highest possible quality of the studies. An important aspect of quality assurance is identification and elimination of various artifacts, that require careful assessment of quality of each study. One of such artifacts, which is the subject of this paper, can be caused by small activity of the radiopharmaceutical remaining at the site of its administration, that in some circumstances can enter the field of view of Discovery NM 530c gamma camera. Such artifacts in the material assessed in this study were observed only in studies conducted in prone position. For this reason, the material consisted only of male patients, since in our department female patients routinely only undergo the study in supine position. During our work with the Discovery NM 530c camera, the artifact was also occasionally observed in patients examined in the supine position, but this occurred much less frequently than in the prone position. In studies carried out in supine position, arms of the patient are placed on a support above the head, which usually positions the elbows and forearms (most common areas of radiopharmaceutical injection) outside the field of view of the camera detectors. Carrying out studies in prone position requires using a different support, on which elbows and forearms are positioned closer to the chest, so radiopharmaceutical injection site is much more likely to enter the field of view of some of the detectors.

The artifact was observed much more frequently in stress studies than in rest studies. This can be explained by the procedure of stress test used in our department, where the cannula used for radiopharmaceutical injection is not removed until the end of image acquisition. The cannula is left for safety reasons, in case of complications after the stress test (for example a sudden drop in blood pressure, sometimes observed after dipyridamole stress test, that could require IV fluids). The valve built into the cannula frequently retains a small amount of administered radiopharmaceutical, that sometimes can still be detected by the camera, even after rinsing the cannula with physiological saline. A small amount of the administered radiopharmaceutical may sometimes leak out of the vein during injection and stay in the surrounding soft tissues, so residual activity can still be present in some cases even after the cannula is removed, but we observe this much less frequently.

In our material, examined artifact had a significant impact on clinical evaluation of MPI studies. Its elimination changed the diagnosis in almost 1/5 of the patients. Images acquired after eliminating the artifact with a shield generally resulted in slightly lower SRS, SSS and SDS values (which means that the artifact tends to generate or increase the extent of radiopharmaceutical uptake defects), but the reverse was also observed (i.e. after placing the shield, previously unobserved perfusion defects became visible). It is unclear how a relatively small activity in injection site, that is located at the edge of the camera field of view, away from the myocardium, can cause such artifacts. Differing impact of these artifacts on images may depend on the location and number of detectors on which the activity in injection site is projected, but the exact mechanism of their generation is unknown.

It seems that the examined artifact can potentially generate false positive results (because the application of the shield most often resulted in a reduction or elimination of the assessed perfusion defects). This can potentially expose the patient to unnecessary invasive diagnostic procedures (coronary angiography), which is associated with an additional dose of ionizing radiation, administration of iodine-based contrast and the risk of complications. It also burdens the health care system with unnecessary costs.

However, it should be emphasized that in a certain group of patients the opposite trend was observed—after the application of the shield, previously invisible, significant perfusion defects were revealed. Such potentially false negative results were observed less frequently, but they are more dangerous for patients. As a result, a patient with severe CAD who most likely requires revascularization and should be referred for coronary angiography, may be misdiagnosed and referred only for observation or conservative treatment. This may lead to deterioration of the health of the patient or even death due to a myocardial infarction.

Due to the above observations, eliminating the artifact is necessary for the correct assessment of MPI studies. In our work, a specially designed lead shield was used for this purpose, which was placed on the arm of the patient, covering the injection site. Such shield is simple in design and cheap (it was created based on a commercially available thyroid shield), and its application is quick and easy and does not affect the course of the examination or the comfort of the patient. Therefore, its routine use in every patient examined with the Discovery NM 530c camera can be considered, especially in the case of studies conducted in the prone position.

Some alternative methods of eliminating the assessed artifacts are also worth considering. One simple way to eliminate them is to change the location of radiopharmaceutical administration. The field of view of the camera includes only the right elbow and a fragment of the right arm. Administering radiopharmaceutical in the distal part of the right arm or anywhere on the left arm makes it practically impossible for the injection site to enter the field of view of the camera, so it should prevent the occurrence of such artifacts even if small residual activity is left there. Removing the cannula before image acquisition also could significantly reduce the occurrence of such artifacts. However, due to safety reasons, this may not be applicable to stress studies. Moreover, it does not guarantee the elimination of artifacts—they can still occasionally occur when some radiopharmaceutical activity leaks outside the vein. Another way to minimize the occurrence of these artifacts is performing the studies only in supine position. Different placement of arms of the patient in this position in most cases (although not always) removes the injection site from the field of view of the camera. This is not an optimal solution for male patients however, where prone position mitigates other frequently observed artifacts affecting the inferior wall (caused by attenuation of radiation by the soft tissues of the abdomen). The last possible method of eliminating such artifacts is software-based masking. Xeleris software enables editing and masking acquired raw images before reconstruction, which could be used to remove the spot of activity visible in the injection site. This process is quite time-consuming however, and the impact of such modifications on reconstructed images is not known. Software-based elimination of such artifacts could be a useful tool to apply retrospectively, in cases where the artifact is found after image acquisition is finished and can no longer be properly eliminated, so the evaluation of this method will be the subject of further studies.

The limitation of our study is lack of robust clinical verification of the MPI results in other imaging modalities. Most patients either did not have current angio-CT or coronarography before our study or had studies with inconclusive results (which were an indication for the follow-up MPI). Most patients also did not have indications for such studies after MPI, as in 1 year follow-up only 5 patients underwent coronary angiography and none had angio-CT.

Additionally, no cases of artifacts in women or patients examined in supine position were found during the enrollment of patients for this study, likely due to their very rare occurrence. Hence their exact impact on study assessment is not known, but it is reasonable to recommend preventing or eliminating them when possible as well.

## Conclusions

1. Radiopharmaceutical activity remaining in the administration site may generate artifacts in myocardial perfusion images acquired using Discovery NM 530c camera, especially in patients examined in prone position;
2. A shield with a lead equivalent thickness of approximately 2.5 mm is sufficient to cover residual activity and eliminate above mentioned artifacts;
3. Elimination of these artifacts changes the final study assessment in almost 20% of patients, most often by eliminating previously visible stress-induced perfusion defects;

## Supplementary Information

Supplementary material 1.

## Data Availability

The datasets generated and analyzed during the current study are included in this published article and its supplementary information files.

## Abbreviations

CAD: Coronary artery disease
MPI: Myocardial perfusion imaging
SSS: Summed stress score
SRS: Summed rest score
SDS: Summed difference score
SPECT: Single photon emission computed tomography
PET: Positron emission tomography
